# The RNA binding protein HuR does not interact directly with HIV-1 reverse transcriptase and does not affect reverse transcription in vitro

**DOI:** 10.1186/1742-4690-7-40

**Published:** 2010-05-07

**Authors:** Jinwoo Ahn, In-Ja L Byeon, Sanjeewa Dharmasena, Kelly Huber, Jason Concel, Angela M Gronenborn, Nicolas Sluis-Cremer

**Affiliations:** 1 Department of Structural Biology, Division of Infectious Diseases, University of Pittsburgh School of Medicine, Pittsburgh, PA 15261, USA; 2 Department of Medicine, Division of Infectious Diseases, University of Pittsburgh School of Medicine, Pittsburgh, PA 15261, USA

## Abstract

**Background:**

Lemay *et al *recently reported that the RNA binding protein HuR directly interacts with the ribonuclease H (RNase H) domain of HIV-1 reverse transcriptase (RT) and influences the efficiency of viral reverse transcription (Lemay *et al*., 2008, Retrovirology 5:47). HuR is a member of the embryonic lethal abnormal vision protein family and contains 3 RNA recognition motifs (RRMs) that bind AU-rich elements (AREs). To define the structural determinants of the HuR-RT interaction and to elucidate the mechanism(s) by which HuR influences HIV-1 reverse transcription activity *in vitro*, we cloned and purified full-length HuR as well as three additional protein constructs that contained the N-terminal and internal RRMs, the internal and C-terminal RRMs, or the C-terminal RRM only.

**Results:**

All four HuR proteins were purified and characterized by biophysical methods. They are well structured and exist as monomers in solution. No direct protein-protein interaction between HuR and HIV-1 RT was detected using NMR titrations with ^15^N labeled HuR variants or the ^15^N labeled RNase H domain of HIV-1 RT. Furthermore, HuR did not significantly affect the kinetics of HIV-1 reverse transcription *in vitro*, even on RNA templates that contain AREs.

**Conclusions:**

Our results suggest that HuR does not impact HIV-1 replication through a direct protein-protein interaction with the viral RT.

## Background

Reverse transcription of the viral single-stranded (+) RNA genome into double-stranded DNA is a critical step in the HIV-1 life-cycle. Although the viral proteins nucleocapsid, matrix, integrase, tat, nef and vif may participate in the regulation and/or efficiency of reverse transcription [1-6], synthesis of the nascent HIV-1 DNA is entirely carried-out by the DNA polymerase and ribonuclease H (RNase H) activities of HIV-1 reverse transcriptase (RT). HIV-1 RT is an asymmetric heterodimer composed of 66 kDa (p66) and 51 kDa (p51) subunits [7]. The p66 subunit can be subdivided into DNA polymerase, connection and RNase H domains. The p51 subunit is derived from p66 by HIV-1 protease cleavage of the C-terminal RNase H domain. The p66/p51 HIV-1 RT heterodimer contains one DNA polymerization active site and one RNase H active site, which both reside in the p66 subunit in spatially distinct regions [7].

Recent studies suggest that host cell proteins may also play an important role in the timing and efficiency of HIV-1 reverse transcription [8-12]. For example, a genome-wide siRNA analysis conducted by König *et al *identified ~30 host cell factors that directly influence either the initiation or kinetics of reverse transcription [8]. However, by its nature, this study did not distinguish direct physical interactions from indirect effects between these host cell factors and any of the viral proteins present in the reverse transcription complex in infected cells. By contrast, in other reports, several host cell proteins, such as HuR, AKAP149 and TRIM37 have all been implicated in direct contacts with HIV-1 RT that impact viral replication [9,10,12]. Validation and comprehensive analysis of these putative RT-host protein interactions are important for a thorough understanding of viral replication and for drug discovery efforts that target HIV-host protein interactions.

**Figure 1:**
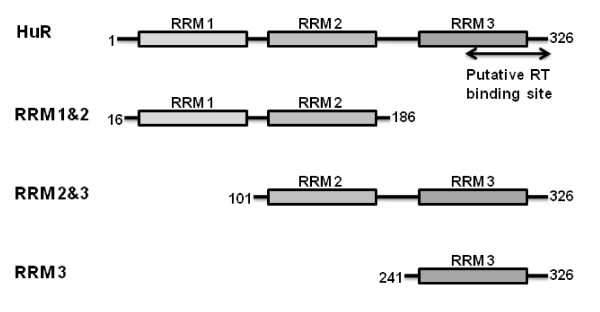
**Schematic representation of HuR constructs used this study**. The three RRM domains are depicted by boxes and the numbers refer to amino acid positions in the full length proteins. The putative binding site for HIV-1 RT is indicated by the arrow at the C-terminus of HuR [9].

The present study was devised to structurally characterize the interaction between HuR and HIV-1 RT that was recently described by Lemay *et al *[9], who identified HuR and HIV-1 RT association in a yeast two-hybrid screen and confirmed the interaction by a homogenous time-resolved fluorescence binding assay. The authors mapped the HIV-1 RT-HuR binding sites to the RNase H domain of RT and to the C-terminus of HuR (see Fig. 1). Importantly, siRNA knockdown of HuR expression in HeLa P4.2 cells was reported to greatly impair both the early and late steps of viral reverse transcription. To further define the structural determinants of the HIV-1 RT-HuR interaction at the atomic level and to elucidate the mechanism(s) by which HuR influences HIV-1 reverse transcription activity *in vitro*, we prepared and characterized four HuR protein constructs and investigated their RT interaction by biophysical methods. We did not find any evidence for a direct interaction between HIV-1 RT and HuR by NMR chemical shift mapping in the ^1^H,^15^N-heteronuclear single quantum coherence (HSQC) spectra of ^15^N-labeled HuR or ^15^N-labeled HIV-1 RT RNase H upon titration with unlabeled RT or HuR. Furthermore, HuR did not affect the kinetics of HIV-1 reverse transcription *in vitro*. Taken together, our results suggest that HuR does not impact HIV-1 replication through a direct interaction with the viral RT.

## Results

### Purification and characterization of HuR

HuR belongs to the Hu family of mRNA stabilizing proteins that interact with AU-rich elements (ARE), sharing significant sequence similarity with the *Drosophila *RNA-binding protein ELAV (embryonic lethal abnormal vision) [13]. The 326 amino acid protein contains three RNA recognition motifs (RRMs), two in the N-terminal half and a third at the C-terminus, separated by a basic ~60 residue linker region (Fig. 1). HuR recognizes a core element of 27 nucleotides in the RNA that contain AUUUA, AUUUUA and AUUUUUA motifs [14-16]. RRM 1 and RRM 2 contribute most of the binding energy in HuR-ARE complex formation [14], while RRM 3 may be responsible for cooperative assembly of HuR oligomers on RNA [17]. In addition, RRM 3 of HuR was reported by Lemay *et al *to directly interact with the RNase H domain of HIV-1 RT [9].

**Figure 2:**
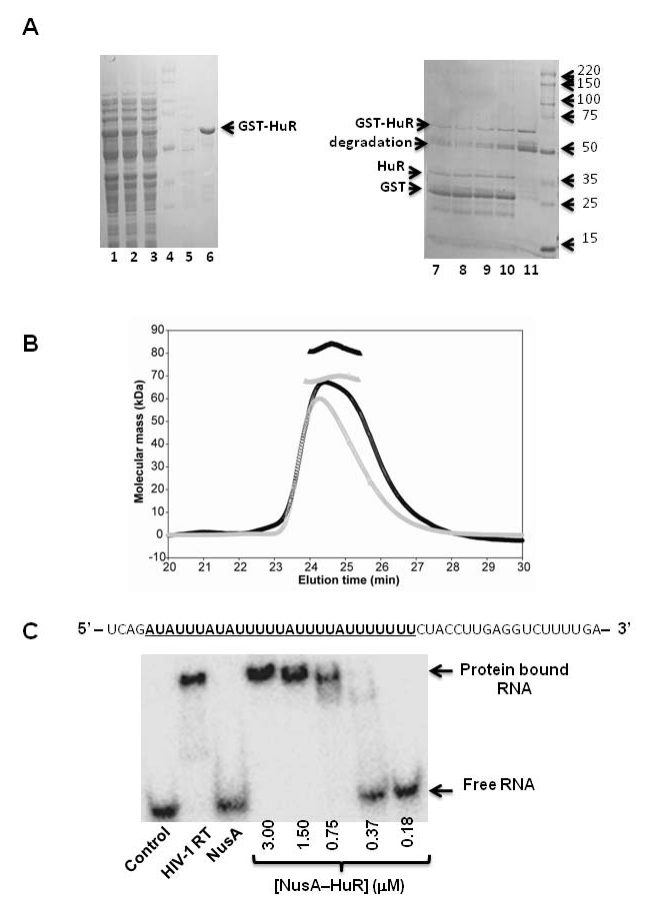
**Purification and biophysical characterization of GST- and NusA-HuR**. **(A) **Analysis of the expression and purification of GST-HuR by SDS PAGE. Lanes 1 and 2 illustrate total protein and total soluble protein after *E. coli *cell lysis, respectively, lane 3 shows the flow-through fraction of the glutathione sepharose column, lane 4 contains molecular weight markers and lane 5 is the eluate during the column wash. Purified GST-HuR eluted from the glutathione sepharose column with 20 mM glutathione is shown in lane 6 and lanes 7-10 contain purified GST-HuR that was incubated with 0.15, 0.1, 0.05 and 0.03 units of thrombin, respectively, for 1 h at room temperature. Lane 11 contains purified GST-HuR incubated for 1 h in buffer at room temperature in the absence of thrombin. **(B) **Size-exclusion multi-angle light scattering analysis of NusA-HuR (black) and NusA-RRM 3 (grey). The elution profiles (circles) and the predicted molecular masses obtained from the light-scattering measurements (triangles) are shown. Both proteins were found to exist > 90% as monomers in solution. **(C) **Gel-shift assay of HuR binding to single-stranded RNA. The sequence of the RNA used in this experiment is shown above the autoradiograph. The ARE sequence is highlighted and underlined. HIV-1 RT and NusA were included as controls in this experiment.

To further define the structural determinants of the HuR-RT interaction and to elucidate the mechanism(s) by which HuR influences HIV-1 reverse transcription activity *in vitro*, we prepared HuR N-terminal fusion proteins with glutathione S-transferase (GST) and NusA. The GST-HuR fusion protein was unstable after purification and underwent substantial degradation at room temperature (Fig. 2A). In contrast, the NusA-HuR fusion was stable and was used in the NMR and HIV-1 RT DNA synthesis reactions described below. We also cloned the RRM 1&2, RRM 2&3, or RRM 3 domains of HuR as N-terminal fusion proteins with NusA (Fig. 1). These domain constructs exhibited sufficient stability after cleavage by TEV protease and removal of the NusA tag for structural characterization by NMR.

The quaternary states of NusA-HuR and NusA-RRM 3 were assessed by multi-angle light scattering (Fig. 2B). Both proteins were found to exist as monomers in solution and the molecular masses of NusA-HuR and NusA-RRM 3 were determined as 82.2 and 68.9 kDa, respectively. These values are within ± 15% of the predicted masses of 99 kDa for NusA-HuR and 73 kDa for NusA-RRM 3. We did not find any evidence for HuR dimer formation, in contrast to a previous report that suggested HuR homodimerization prior to RNA binding [18]. Furthermore, we show that NusA-HuR binds a synthetic RNA template that contains AREs (Fig. 2C), indicating that the fusion does not interfere with the RNA binding activity of HuR.

### Probing the interaction between HIV-1 RT and HuR by NMR

**Figure 3:**
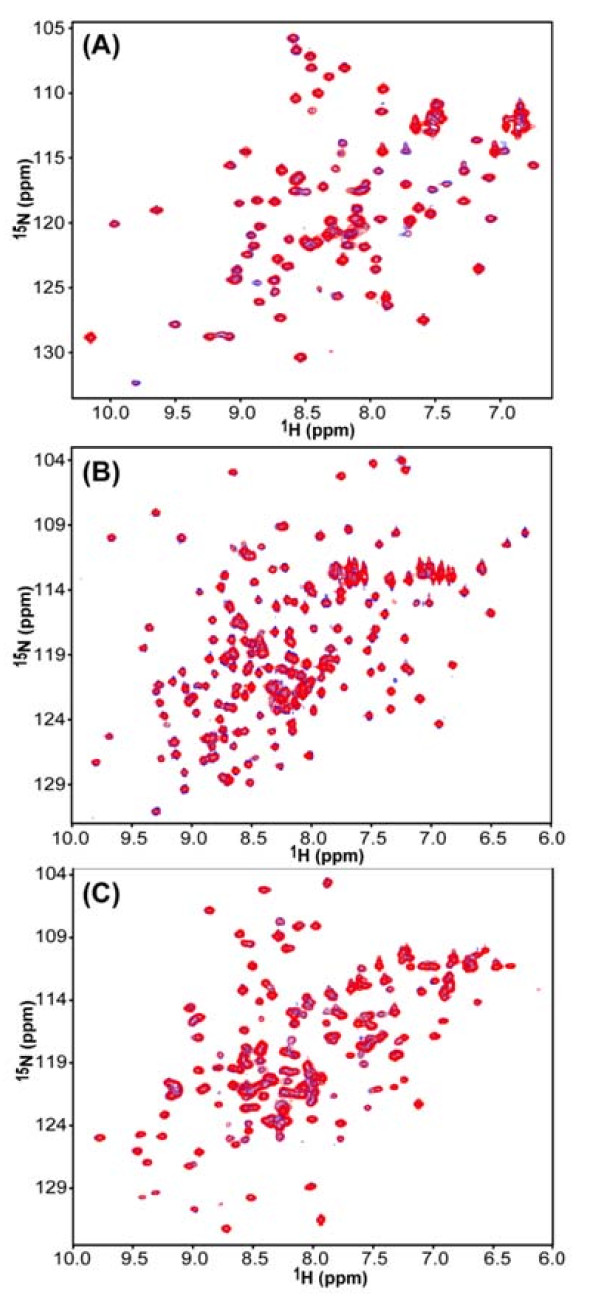
**^1^H,^15^N-HSQC NMR analyses to probe for binding between HIV-1 RT and HuR**. **(A) **Superimposed ^1^H,^15^N-HSQC spectra of 30 μM of the [^15^N]-labeled RRM 3 domain of HuR in the absence (blue) and presence (red) of 60 μM unlabeled full-length HIV-1 RT. **(B) **Superimposed ^1^H,^15^N-HSQC spectra of 200 μM of the [^15^N]-labeled RRM 1&2 domains of HuR in the absence (blue) and presence (red) of 400 μM of the unlabeled HIV-1 RT RNase H domain. **(C) **Superimposed ^1^H,^15^N-HSQC spectra of 60 μM of the [^15^N]-labeled HIV-1 RT RNaseH domain in the absence (blue) and presence (red) of 60 μM of unlabeled NusA-HuR.

We used ^1^H,^15^N-HSQC NMR spectroscopy [19,20] to probe whether a direct interaction between HIV-1 RT and HuR could be identified *in vitro*. Several different proteins were investigated: (i) the ^15^N-labeled RRM 3 domain of HuR was titrated with full-length HIV-1 RT (Fig. 3A); (ii) the ^15^N-labeled RRM 1&2 of HuR was titrated with the RNase H domain of HIV-1 RT (Fig. 3B); and (iii) the ^15^N-labeled RNase H domain of HIV-1 RT was titrated with NusA-HuR (Fig. 3C). All three ^15^N-labeled proteins exhibited well-dispersed ^1^H,^15^N-HSQC spectra, indicative of well-folded, stable structures. No changes in their ^1^H,^15^N-HSQC spectra were observed upon titration with the unlabelled binding partners up to a two-fold molar excess. It, therefore, is highly unlikely that direct protein-protein contacts are present for any of the above protein pairs.

### HuR does not impact the DNA synthesis efficiency of HIV-1 RT *in vitro*

**Figure 4:**
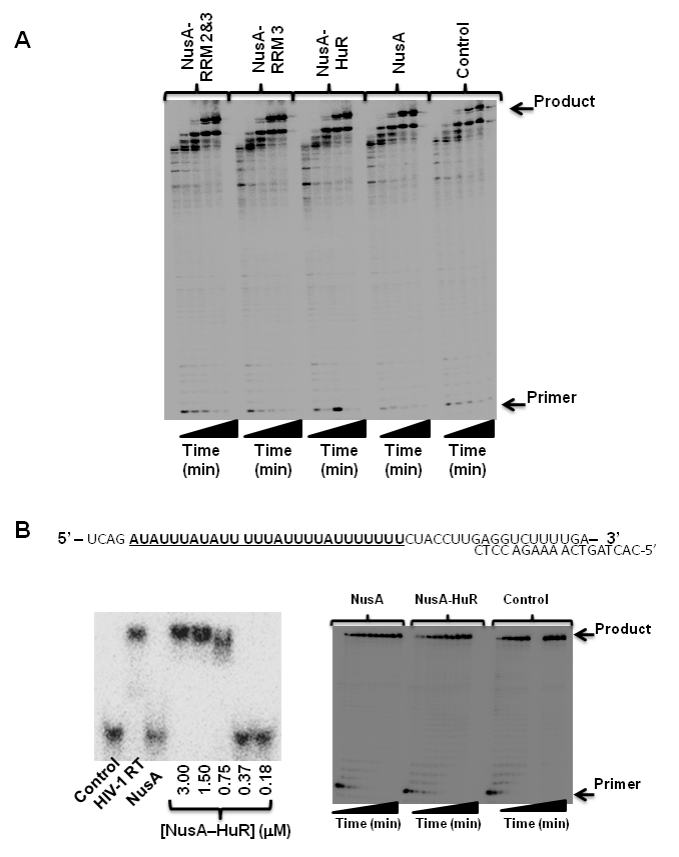
**Steady-state DNA synthesis by HIV-1 RT in the presence of NusA-HuR**. **(A) **HIV-1 RT DNA synthesis on a heteropolymeric RNA template corresponding to the HIV-1 sequence of (-) strong stop DNA. The 18 nucleotide DNA oligonucleotide primer is complementary to the HIV-1 tRNA^Lys3 ^primer binding site in the RNA template. The reaction was carried out for 2, 5, 10, 30 60 min, respectively. **(B) **HIV-1 RT DNA synthesis carried out on an ARE containg T/P substrate. The sequences of the T/P is indicated with the ARE in bold and underlined. Binding of NusA-HuR to this T/P is observed (left-hand autoradiograph). However, the efficiency of HIV-1 RT DNA synthesis is not significantly affected (right-hand autoradiograph). The reaction times for DNA synthesis were 0, 1, 2, 3, 4, 5, 7.5, 10, 15, 20 and 30 min, respectively.

Although our NMR data exclude the presence of direct physical protein-protein contacts between HIV-1 RT and HuR, indirect effects from one protein to the other may occur, possibly mediated by RNA. To investigate this possibility, we carried out HIV-1 RT DNA synthesis reactions using two different template/primer (T/P) substrates. In the first, we used a long heteropolymeric RNA template, corresponding to the HIV-1 sequence used for (-) strong stop DNA synthesis, that was primed with an 18 nucleotide DNA primer. In this assay, 173-nucleotide incorporation events are needed to produce the full-length DNA product, allowing multiple dNTP additions [21,22]. Importantly, this template does not contain AREs that would interfere with HuR binding to the RNA (data not shown). DNA synthesis reactions carried out with this T/P in the presence of NusA-HuR, NusA-RRM 3 or NusA-RRM 2&3 were not significantly different from the control reaction in the presence of NusA only (Fig. 4A). Next, we investigated HIV-1 RT DNA synthesis on a T/P substrate that contains AREs (Fig. 4B). Gel-shift assays confirmed that HuR bound to this T/P (Fig. 4B). However, the binding of HuR to the RNA did not appear to significantly affect the efficiency of HIV-1 RT reverse transcription on this T/P substrate either (Fig. 4B).

## Discussion

Lemay *et al *identified HuR as a binding partner for HIV-1 RT in a yeast two-hybrid screen of a random primed cDNA library derived from CEMC7 lymphocytes, and a physical interaction *in vitro *was proposed based on time-resolved fluorescence data [9]. In the reported fluorescence experiment, serial dilutions of GST-HuR (or GST alone) were incubated with a constant amount of C-terminal hexahistidine tagged HIV-1 RT for 24 hours at 4°C. Subsequently, the interaction was probed by fluorescence energy transfer using anti-GST antibodies conjugated with the donor TBPEu^3+ ^and anti-hexahistidine antibodies conjugated with the acceptor XL665. In our experiments, we discovered that the GST-HuR fusion protein is not stable in solution for extended periods and is subject to degradation, casting doubt on the validity of the above interpretation. Indeed, Lemay *et al. *may have looked at an interaction, most-likely a non-specific one, between HIV-1 RT and a degraded/unfolded form of GST-HuR. In this regard it is well known that marginally stable proteins are prone to aggregation, a non-specific protein-protein interaction. It should be noted, however, that Lemay *et al *cloned HuR into the pGEX-4T-1 vector whereas we cloned it into the pGEX-2T vector. The resultant fusion proteins are identical in amino acid sequence except that the Lemay *et al *construct contains an additional proline residue located between the thrombin cleavage site and N-terminus of HuR. Although unlikely, this minor difference could contribute to differences in the relative solution stabilities of the GST-HuR constructs used in the two different studies.

Lemay *et al. *also reported that knockdown of HuR expression in HeLa P4.2 cells by RNA interference inhibited both the early and late steps of HIV-1 reverse transcription. While this could be caused by a direct effect of HuR on reverse transcription, it also could arise via indirect effects on other host cell factors that are important in HIV-1 replication. For example, it is well documented that tumor-necrosis factor-alpha (TNF-α) levels in cells significantly impact HIV-1 replication [23-26] and that the expression of many inflammatory cytokines, including TNF-α, is tightly regulated at the post-transcriptional level by HuR [27]. Interestingly, several studies have demonstrated that HuR may undergo post-translational phosphorylation at S202 and/or S242 [28,29]. Therefore, one cannot rule out the possibility that Lemay *et al *identified an interaction between a post-translationally modified HuR protein and HIV-1 RT in their yeast-two hybrid screen, and that this interaction may be of biological relevance.

In summary, the NMR chemical shift titration experiments with purified proteins, presented in this report, demonstrate unambiguously that no direct protein-protein interactions between HIV-1 RT and HuR are present *in vitro *up to concentrations of ~200 μM. It should be noted that the RT used in our study is derived from an LAI isolate (group M, subtype B), whereas the RT used in the Lemay *et al *study was derived from a BH10 isolate (group M, subtype B). There are amino acid differences between these two isolates in their RNase H domains at codons 447 [N (BH10) → S (LAI)], 461 (K → R), 468 (P → T), 471 (N → D), 482 (Y → H) and 559 (V → I). However, all of these substitutions exist as polymorphisms in the RT subtype B sequences deposited in the Stanford HIV database. Furthermore, although we found no evidence for a direct protein-protein interaction between HuR and HIV-1 RT in this study, an indirect interaction may be mediated by RNA. However, we could not detect any influence of HuR on HIV-1 RT DNA synthesis, even on T/P substrates that contain AREs and bound both HIV-1 RT and HuR (Fig. 4B). Therefore, our results suggest that HuR does not interfere with HIV-1 replication through a direct interaction with the viral reverse transcription complex, but through indirect effects possibly mediated via unidentified host factors and/or RNA.

In the search for host-pathogen interactions, a burgeoning field in modern virology, many potential interactions have been identified for HIV-1 in the last 2 or 3 years through high through-put screens [8-10,12,30,31]. Our present follow up study using purified proteins illuminates some of the potential pitfalls associated with such approaches, and highlights the urgent need to carry out stringent biophysical validation of any putative interaction.

## Methods

### Cloning

The cDNA encoding HuR (National Center for Biotechnology Information Reference Sequence NM_001419) was purchased from Open Biosystems (Rockford, IL) and from Origene Technologies (Rockville, MD). DNA encoding full-length HuR (residues 1-326) was cloned between the EcoR1 and Xho1 restriction sites of pET43A (EMD Chemicals Inc., San Diego, CA) and between the BamH1 and EcoR1 restriction sites of pGEX-2T (GE Healthcare, Piscataway NJ). A TEV protease recognition sequence (ENLYFQS) was engineered at the C-terminus of the NusA fusion protein in pET43A. Constructs coding for both RRM 1&2 (residues 16-186) and RRM 3 (residues 241-326) domains of HuR were cloned between the EcoRI and XhoI restriction sites of pET21a (EMD Chemicals Inc., San Diego, CA). The coding sequence for the RNase H domain of RT (residues 433-560) was amplified and cloned between EcoRI and XhoI restriction sites of pET32a (EMD Chemicals Inc., San Diego, CA) and was modified to include a TEV Protease recognition site at the C-terminus of thioredoxin [32]. The integrity of all clones was assessed by full-length sequencing of the respective plasmids.

### Protein expression and purification

The HuR constructs as well as the RNase H domain of HIV-1 RT were expressed in *E. coli *Rosetta 2 (DE3), cultured in Luria-Bertani media. Protein expression was induced by the addition of 0.4 mM IPTG, and the cells were grown at 18°C for 16 to 20 h. Cells were opened using a microfluidizer (Newton, MA), and proteins were purified using 5 mL Ni-NTA columns. Aggregated material was removed by gel-filtration column chromatography using Hi-Load Superdex200 16/60 (GE Healthcare, Piscataway, NJ) equilibrated with a buffer containing 25 mM sodium phosphate, pH 7.5, 150 mM NaCl, 10% glycerol, 1 mM DTT, and 0.02% sodium azide. NusA-HuR and NusA-RRM 3 were further purified on a Hi-Trap QP column (GE Healthcare, Piscataway, NJ) at pH 7.5 using a 0-1 M NaCl gradient. NusA-RRM 1&2 was further purified on a Hi-Trap SP column (GE Healthcare, Piscataway, NJ) at pH 6.5 using a 0-1 M NaCl gradient. The RNase H domain of HIV-1 RT was obtained after TEV protease digestion of the TRX fusion protein and purified on a Hi-Trap SP column (GE Healthcare, Piscataway, NJ) at pH 7.5 using a 0-1 M NaCl gradient. Buffer exchange was carried out using Amicon concentrators (Millipore, Billerica, MA), and proteins were stored at 4°C in solution. GST-HuR and HIV-1 RT were purified as described previously [9,33,34]. For isotopic labeling, proteins were expressed as described above in modified minimal media using ^15^NH_4_Cl as the sole nitrogen source. The [^15^N]-labeled HuR RRM 3 and HuR RRM 1&2 used in NMR experiments contained extra amino acids (LEHHHHHH) at their C-termini, while the [^15^N]-labeled HIV-1 RT RNase H domain NMR sample contained two additional amino acids (EF) at its N-terminus.

### Multi-angle light scattering

Light-scattering data were obtained using an analytical Superdex-200 column (1 cm × 30 cm) with in-line multi-angle light-scattering (DAWN HELEOS, Wyatt Technology, Inc., Santa Barbara, CA) and refractive index detectors (OPTILAB DSP, Wyatt Technology, Inc.). Proteins were applied to the pre-equilibrated column at a flow-rate of 0.5 ml/min at room temperature and eluted with 25 mM sodium phosphate buffer, pH 7.5, containing 150 mM NaCl, 10 mM β-mercaptoethanol, 0.02% sodium azide and 5% glycerol. Total protein amounts loaded were 100 μL of 7.8 mg/mL NusA-RRM 3 and 100 μL of 1.1 mg/mL NusA-HuR.

### NMR spectroscopy

For the NMR experiments, 30-200 μM uniformly- [^15^N]-labeled protein samples without and with equimolar or two-fold molar amounts of the proposed binding partner were prepared using the identical buffer (25 mM sodium phosphate buffer, pH 7.5, containing 150 mM NaCl, 10 mM β-mercaptoethanol, 0.02% sodium azide, 5% glycerol and 7% ^2^H_2_O). All ^1^H,^15^N-HSQC NMR experiments [16] were performed at 17°C on a Bruker Avance 600 MHz spectrometer, equipped with a 5 mm triple resonance and z-axis gradient cryoprobe.

### Gel Mobility Shift Assays

Gel mobility shift assays were used to evaluate the binding interaction between HuR and RNA. In these assays, the amount of RNA-bound HuR present in solution is assessed by native gel electrophoresis. HuR (0-3 μM total) was equilibrated with 100 nM of ^32^P-labeled RNA for 1 hr in 50 mM Tris pH 7.5, 50 mM KCl at 37°C. Samples were then loaded on a 7% polyacrylamide gel in 40 mM Tris-acetate, pH 8.0, containing 1 mM EDTA. Gels were run at room temperature for 30 min (100 V constant voltage), and radioactivity was quantified using a Bio-Rad GS525 Molecular Imager (Bio-Rad Laboratories, Inc., Hercules, CA).

### HIV-1 RT DNA synthesis reactions

The heteropolymeric RNA-dependent DNA polymerase T/P corresponding to the HIV-1 sequence used for (-) strong stop DNA synthesis was prepared as described previously [21,22]. The 18 nucleotide DNA oligonucleotide primer used in this experiment is complementary to the HIV-1 tRNA^Lys3 ^primer binding site and was 5'-end radiolabelled with γ- [^32^P]-ATP prior to annealing to the RNA template. DNA polymerization reactions were carried out by incubating 50 nM HIV-1 RT with 3 μM NusA-HuR in 50 mM Tris-HCl (pH 8.0), 50 mM KCl for 5 min before the addition of 20 nM T/P, containing 1 μM dNTP and 10 mM MgCl_2_. After defined incubation periods, aliquots were removed and the reaction was quenched with equal volumes of gel loading dye. Products were separated by denaturing gel electrophoresis and radioactivity was quantified with a Bio-Rad GS525 Molecular Imager.

## Competing interests

The authors declare that they have no competing interests.

## Authors' contributions

Conceived and designed the experiments: JA, IJLB, AMG and NSC. Performed the experiments: JA, IJLB, SD, KH and JC. Analyzed the data: JA, IJLB, AMG and NSC. Wrote the paper: JA, IJLB, AMG and NSC.
